# A 3D Optical Surface Profilometer Using a Dual-Frequency Liquid Crystal-Based Dynamic Fringe Pattern Generator

**DOI:** 10.3390/s16111794

**Published:** 2016-10-27

**Authors:** Kyung-Il Joo, Mugeon Kim, Min-Kyu Park, Heewon Park, Byeonggon Kim, JoonKu Hahn, Hak-Rin Kim

**Affiliations:** School of Electronics Engineering, Kyungpook National University, Daegu 41566, Korea; kijoo@knu.ac.kr (K.-I.J.); im2781@gmail.com (M.K.); mkpark@ee.knu.ac.kr (M.-K.P.); heewonpark@ee.knu.ac.kr (H.P.); bgkim@knu.ac.kr (B.K.); jhahn@knu.ac.kr (J.H.)

**Keywords:** optical surface profilometry, interference, phase modulation, liquid crystal, dynamic fringe pattern generator

## Abstract

We propose a liquid crystal (LC)-based 3D optical surface profilometer that can utilize multiple fringe patterns to extract an enhanced 3D surface depth profile. To avoid the optical phase ambiguity and enhance the 3D depth extraction, 16 interference patterns were generated by the LC-based dynamic fringe pattern generator (DFPG) using four-step phase shifting and four-step spatial frequency varying schemes. The DFPG had one common slit with an electrically controllable birefringence (ECB) LC mode and four switching slits with a twisted nematic LC mode. The spatial frequency of the projected fringe pattern could be controlled by selecting one of the switching slits. In addition, moving fringe patterns were obtainable by applying voltages to the ECB LC layer, which varied the phase difference between the common and the selected switching slits. Notably, the DFPG switching time required to project 16 fringe patterns was minimized by utilizing the dual-frequency modulation of the driving waveform to switch the LC layers. We calculated the phase modulation of the DFPG and reconstructed the depth profile of 3D objects using a discrete Fourier transform method and geometric optical parameters.

## 1. Introduction

Recently, the demand for acquiring 3D information has increased, accompanied by improvements in several 3D applications like 3D displays, 3D printing technologies, 3D medical or dental imaging systems, and 3D vision modules for robot or vehicle applications [1,2,3,4,5,6]. For these applications, 3D depth information together with conventional 2D images is critically needed, and cannot be obtained with conventional 2D vision systems. Optical 3D vision systems can measure 3D information for an object with a wide scope in a relatively short time because they obtain the 2D coordinate information together with the depth information optically in parallel using a Charge-Coupled Device (CCD) or Complementary Metal Oxide Semiconductor (CMOS) image sensor.

The optical 3D surface profilometer can optically measure the surface morphology of an object and compute the depth information without direct contact. In general, optical surface profilometers, which extract 3D depth information from distortions of the projected optical beam patterns, need an optical beam pattern generator module to utilize several structured beam. To measure a surface profile of 3D objects, N-bits of binary-coded stripe patterns could be projected [7], where the number of the binary-coded projection beam patterns needed to be increased to improve the measurement resolution. By utilizing gray-level beam patterns, the number of projection patterns could be effectively reduced [8], but this approach needs more complex spatial light modulators (SLMs) with a higher pixel resolution and a larger pixel density to generate the gray-level patterns. To effectively reduce the measurement time, a one-shot scanning method using periodic grid patterns was proposed [9]. Using a coherent light source, interference fringe patterns could be projected [10,11,12,13,14]. To avoid the optical phase ambiguity from the fringe patterns distorted by the surface morphology, multiple sets of interference patterns were needed, and phase unwrapping or geometrical parameter methods were applied to reconstruct surface depth profiles. The depth extraction method based on the geometrical parameters could provide the depth and position value informations in the absolute coordinate system without the phase unwrapping process [10]. These optical techniques do not damage the surface morphology of an object even for a soft surface.

In most cases of optical surface profilometries, to acquire more precise 3D depth information, several sets of specific beam patterns are needed. These are generated by beam pattern projecting modules such as mechanically moving wedge plates [15], tunable gratings [16], switchable gradient index lenses [17], polymer dispersed liquid crystal (LC) techniques [18,19], and several types of phase modulators using SLMs [20,21]. Recently, to obtain color information of an object in addition to the 3D depth information, time-sequential projection of structured and RGB-colored beam patterns was also proposed using fast switching digital light processing (DLP) projectors [22]. However, conventional projection units based on commercial SLMs and DLP are too bulky and not cost-effective for industrial field applications, especially dental imaging, which needs an elaborate and complex semiconductor manufacturing process for the preparation of backplanes to control the 2D phase or intensity patterns with matrix driving schemes [23,24,25].

In this study, we propose an LC-based dynamic fringe pattern generator (DFPG) for a more compact 3D optical surface profilometer system, which can generate multiple fringe patterns to enhance the 3D depth extraction and avoid the optical phase ambiguity in analyzing the 3D depth profile from distorted fringe patterns induced by the surface morphologies. Sixteen interference patterns are generated with four-step phase shifting and four-step spatial frequency varying schemes by the proposed LC-based DFPG without a mechanically moving part. In our DFPG, the 16 sets of fringe patterns can be generated by a single, compact LC cell, which has one common slit operated by an electrically controllable birefringent (ECB) LC mode and four switching slits operated by a twisted nematic (TN) LC mode. Four different moving fringe patterns are controlled by the phase value of the ECB LC layer behind one common slit. The interferometric fringe patterns with four different spatial frequencies are controlled by electrically selecting one of the switching slits operated by the TN mode. The switching time of the DFPG module, required for projecting the 16 sets of fringe patterns, is minimized by utilizing the dual-frequency-based LC switching scheme. We present the optical and material design of the LC-based DFPG and its manufacturing process obtaining multiple sets of fringe patterns using a compact module with a fast switching time. The electrical switching properties of the phase modulation and the slit spacing controls for the interference patterns of the DFPG are characterized and the 3D depth profile reconstruction from the distorted fringe patterns using the discrete Fourier transform (DFT) method and geometric optical parameters is presented.

## 2. Operation Principle of the DFPG and the Theory of Depth Extraction

### 2.1. Schematic and Operation Principle of the DFPG

#### 2.1.1. Schematic of the DFPG

**Figure 1:**
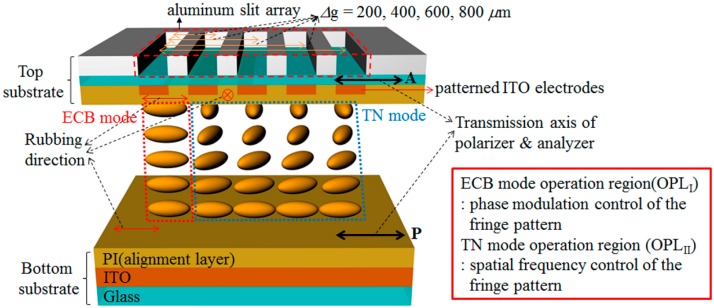
Schematic of the LC-based dynamic fringe pattern generator for projecting multiple sets of interference patterns with phase-shifting and spatial-frequency-varying properties.

In this study, the DFPG was designed to develop a 3D optical surface profilometer that can exhibit multiple sets of interference fringe patterns with four steps of the spatial frequency and four steps of the phase shifting properties without any mechanical translation stages. As shown in Figure 1, the DFPG consists of a multi-directional LC alignment layer on the top substrate to provide the ECB LC and TN LC modes for the phase-shifting common slit and four switching slits, respectively. On the other side of the multi-alignment layer of the top substrate, five Al slits were fabricated by the lift-off process. The indium tin oxide (ITO) layer under the multi-directional alignment layer was also patterned using photolithography to control one ECB LC layer and four TN LC layers individually by applying appropriate voltages to them. The positions of the patterned ITO layer, patterned LC alignment layers, and Al slits were precisely aligned with each other with alignment marks. The spacings between one common slit with the ECB LC mode and the other switching slits with the TN LC modes were Δg = 200, 400, 600, and 800 µm. The widths of the Al slits and the patterned ITO electrodes were 52 and 50 µm, respectively. A uni-directionally rubbed LC alignment layer was prepared on the non-patterned ITO glass substrate as the bottom substrate. Parallel polarizers were attached on both sides of the two substrates.

The multi-spatial frequency properties of the interference fringe patterns were achieved by applying a turn-on voltage on one of the switching slits prepared with the TN LC mode. The four-step phase shifting properties were developed by controlling the applied voltage through the ECB LC mode layer used for the common slit. As a result, the DFPG can generate 16 sets of the multiple fringe patterns that exhibit four different spatial frequencies together with four-step phase-shifted fringe patterns for each selected spatial frequency.

The DFPG was fabricated with a dual-frequency LC (MLC-2048, Merck Ltd., Seoul, Korea) to enhance its switching response time required for generating the 16 sets of fringe patterns. The inversion frequency of the dielectric anisotropy of MLC-2048 is 50 kHz. Its dielectric anisotropy values are Δε = 3.2 at the low frequency AC driving of 1 kHz and Δε = −3.4 at the high frequency AC driving of 100 kHz. The extraordinary refractive index of the dual-frequency LC is 1.7192, and the ordinary refractive index is 1.4978. Therefore, the cell gap of the DFPG was calculated as 2.48 µm to realize over 3π/2 phase modulation of the LC layer. We dropped a mixture of an optical adhesive polymer (NOA 65, Norland Products, Inc., Cranbury, NJ, USA) with ball spacers on the four edges of the patterned ITO/Al substrate, and the top substrate was covered with the bottom electrode substrate. The ball spacers uniformly supported the cell gap of the DFPG required for reliable phase modulation. In our DFPG, the cell gap was about 4 µm. The empty DFPG cell was filled with MLC-2048 by the capillary force over the nematic-isotropic phase transition temperature (*T_NI_* = 106.2 °C) of the LC. After slowly cooling the LC cell to room temperature, the LC layer was well aligned multi-directionally along the patterned rubbing directions of the alignment layer of the top substrate, showing the two LC domains of the ECB and TN LC modes.

#### 2.1.2. Operation Principle of the DFPG

**Figure 2:**
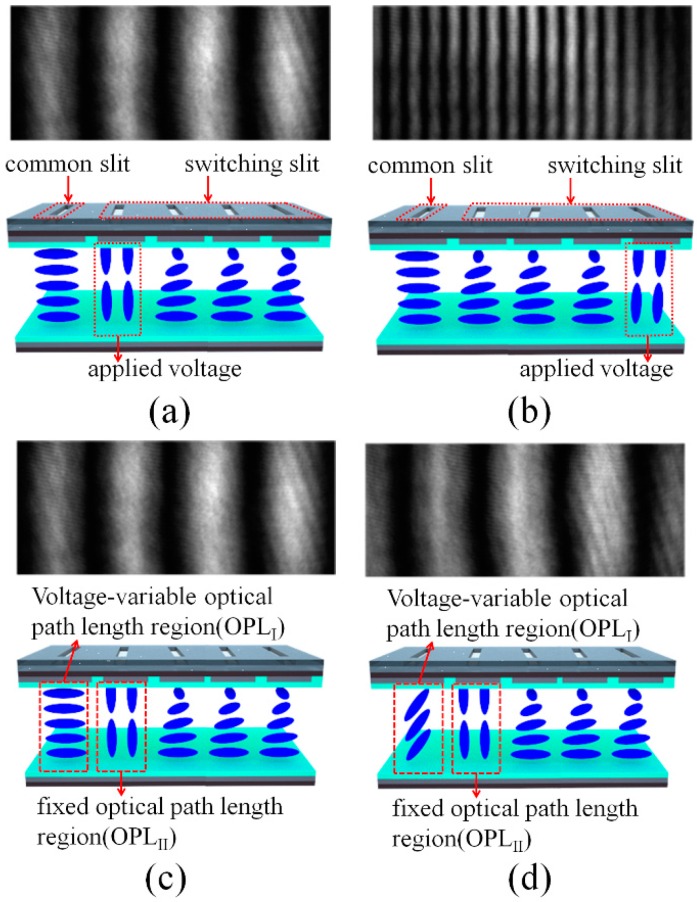
Operation principles of the DFPG switched by field-induced patterned LC orientations and the resulting projected fringe patterns. (**a**,**b**) show the LC orientations and spatial frequency variations of the projected fringe patterns by control of the selected switching slit with applied voltages: (**a**) Δg = 200 µm slit spacing and (**b**) Δg = 800 µm slit spacing; (**c**,**d**) show the field-induced LC orientations and the moving fringe patterns by control of the phase shifting at the common slit with applied voltages under the fixed slit spacing condition (Δg = 200 µm slit spacing).

The operation principles of our DFPG and field-dependent LC orientations on each patterned slit are shown in Figure 2, where the projected fringe patterns, which were measured under the far-field interference conditions using a CCD camera, are co-plotted according to the applied voltage conditions. As shown in Figure 2, the common slit is aligned with the ECB LC mode that can modulate the phase shifting and the four switching slits are aligned with the TN LC mode that can modulate the spatial frequencies of the fringe pattern. Under the parallel polarizer condition, the LC alignment direction of the bottom substrate is parallel with the transmission axes of the top and bottom polarizers. Therefore, the incident polarization state after the bottom polarizer does not change irrespective of the applied voltage within the ECB LC mode. The incident beam passing through the ECB LC layer always transmits the top polarizer after passing though the common slit without intensity loss irrespective of the applied voltage.

However, the polarization states of the incident beams passing through the TN LC layers, initially polarized by the bottom polarizer, are rotated by 90° owing to the polarization rotating effect of the twisted LC structure in the field-off state. Thus, the beams after four switching slits are blocked by the second polarizer without an applied voltage in our parallel polarizer scheme. The dielectric anisotropy of the LC used in our experiment is positive under low frequency AC driving conditions, and the LCs are reoriented along the applied field direction. When a voltage sufficient to fully reorient the LCs along the vertical field direction is applied to the TN LC layer under one of the switching slits by the patterned ITO electrode, the LC molecules in the selected local area can be fully reoriented to the vertically aligned geometry, as shown in Figure 2a,b. Thus, the beam passing through the selected switching slit can be transmitted through the second polarizer. Therefore, depending on the applied voltages of the patterned ITO electrodes under the four switching slits, the distance between two interference slits can be controlled, which enables four different spatial frequencies of the projected fringe patterns, as shown in Figure 2a,b. In Figure 2a,b, the fringe patterns measured under the shortest and the longest slit distances in our DFPG are shown for the example cases of the lowest and the highest spatial frequencies of the projected fringe patterns, respectively. By using these methods, the multi-spatial frequency schemes were developed in the DFPG without any mechanical translation stage part.

Under the fixed spatial frequency attained by selecting the switching slit of one TN LC layer, the four-step phase-shifting schemes can be achieved by applying voltages to the ECB LC layer of the common slit for an appropriate phase difference with the phase of the selected switching slit. The phase shift is decided by the optical path length difference between the ECB LC layer and the field-applied TN LC layer under two slits of one common slit and the selected switching slit, respectively. Figure 2c,d show the initial and the relatively moved fringe patterns according to the applied voltages in the ECB LC layer for the same selected switching slit condition, where the spatial frequencies of two fringe patterns maintain each other. The effective refractive index of the ECB LC layer can be varied by applying voltages because the incident polarization is parallel to the rubbing direction of the LC alignment layer, and the LC layer operated with the ECB LC mode, which has a field-dependent tilting redistribution of the LC layer, does not exhibit any LC twisting distortion irrespective of the applied voltages. Thus, the optical path length passing though the common slit can be modulated according to the voltage applied to the ECB LC layer. For 3D depth extraction from the projected fringe patterns, which are distorted by an object, four-step phase shifting, especially over 3π/2 phase shifting, is needed, and the thickness of the DFPG LC cell is designed considering the birefringence of the LC used in our experiment.

#### 2.1.3. Fabrication Process of the DFPG

**Figure 3:**
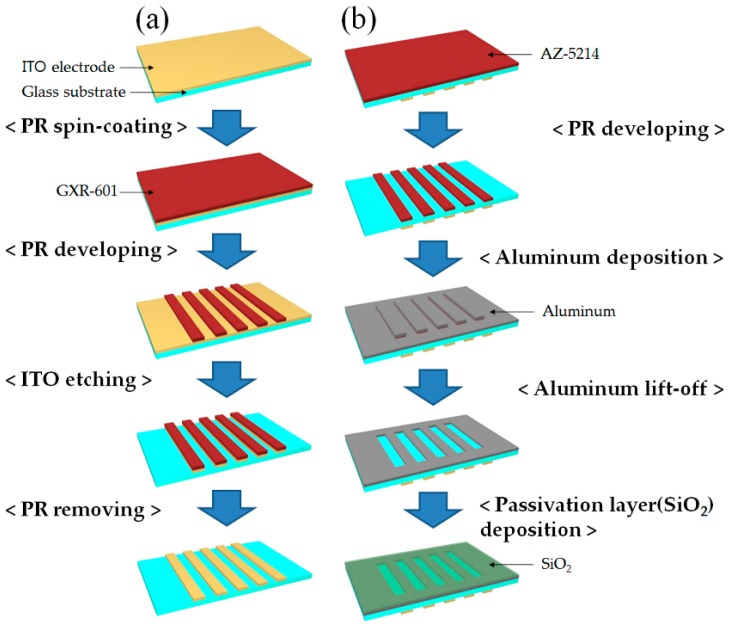
Fabrication process of the top substrate for the multiple slits and the aligned ITO patterns of the LC-based DFPG device: (**a**) ITO electrode patterning and (**b**) Al slit array patterning using the lift-off process.

To electrically control the spatial-frequencies and phase modulation of the fringe patterns, the ITO electrodes were patterned by a photolithography and chemical etching process, as shown in Figure 3a. The first step for developing the patterned ITO electrodes was the spin-coating of a positive photoresistor (GXR-601, AZ Electronic Materials Co., Wiesbaden, Germany) on the ITO glass substrate. The GXR-601 photoresistor was coated under the conditions of 2500 rpm for 5 s, 3500 rpm for 30 s, and 2500 rpm for 5 s. After the spin-coating process, the coated ITO glass was heated to 90 °C on a hotplate for 90 s. Then, the coated photoresistor was projected through the photo-mask pattern by ultraviolet light and was etched with the photoresistor developer (AZ300, AZ Electronic Materials Co.) for 18 s.

The patterned photoresistor coated on the ITO glass was heated again on the hotplate at 120 °C for 3 min. Finally, to define the ITO electrode pattern, the ITO electrode was etched by the ITO etchant (LCE-12K, Cyantek Co., Fort Worth, TX, USA) for 20 min and the photoresistor, which might have remained on the substrate, was removed by an acetone cleaning process.

To generate the interference patterns, the Al slits were prepared on the backside of the patterned ITO surface of the glass substrate. In this study, the Al slits were fabricated using the metal lift-off method to avoid chemical damage on the ITO patterns during our Al slit manufacturing prepared at the same substrate. For the Al slit patterning process, the patterned ITO glass substrate was turned over and the photoresist was patterned with a photo-mask, as shown in Figure 3b. The Al slits must be aligned precisely with the patterned ITO electrodes to control each slit independently. The positions of the patterned ITO electrodes and the Al slits were aligned with the alignment marks. A negative photoresist (AZ-5214, AZ Electronic Materials Co.) was used for the Al slit patterning, which was suitable for the metal lift-off process. After the process of the negative photoresist pattering, the Al layer was deposited using vacuum thermal evaporation equipment with a 500 nm thickness. The Al slit patterns were defined clearly by removing the photoresist with acetone. To protect the Al slits from a physical scratch damage, the SiO_2_ layer was deposited on the patterned Al layer as a passivation layer with a 500 nm thickness.

**Figure 4:**
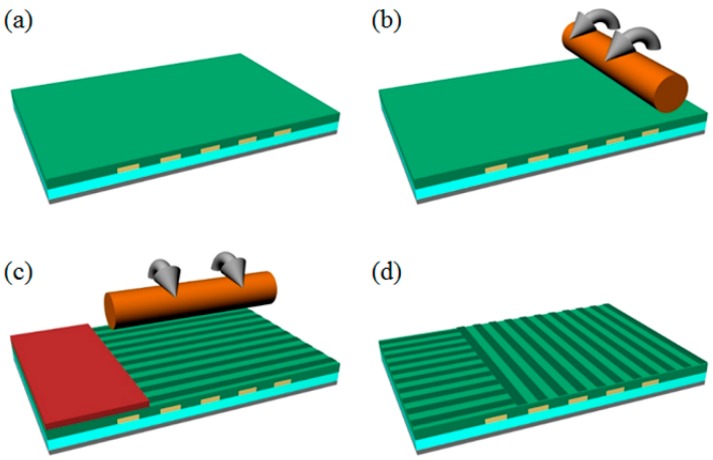
Schematic of the multi-rubbing process for two domains of the initial LC alignments on patterned ITO substrates to enable the ECB LC mode on the common slit for the phase changing of the interference patterns and the TN LC mode on the switching slits for changing the spatial frequency of the projected fringes: (**a**) spin-coating of the LC alignment layer; (**b**) the first rubbing process over the whole area; (**c**) the second rubbing process after the formation of the patterned passivation layer with photoresistor where the rubbing direction is orthogonal to the first rubbing direction; and (**d**) the final multi-rubbed LC alignment layer for the top substrate of the DFPG device, prepared after removing the passivation layer.

To realize the optical 3D surface profilometry system without any mechanical stage, we implemented an orthogonally aligned LC sample, where the two LC alignment directions were precisely aligned with two regions of the common slit and four switching slits. Those were also aligned with the patterned ITO electrodes, as shown in Figure 1 and Figure 4. As shown in Figure 4, the orthogonally aligned LC sample was implemented using the multi-rubbing method on the patterned ITO/Al substrate. After rubbing the LC alignment layer with a soft rubbing cloth attached on the roll-based rubbing machine, the LC molecules can be unidirectionally aligned on the LC alignment surface along the rubbing direction owing to the rubbing-induced surface morphology change and the alignment effect of the LC-interactive side chains of the LC alignment surface material [26]. However, with this conventional rubbing process, the multi-directional patterned LC alignment condition, required in our DFPG for coexisting two LC modes of the ECB LC mode and the TN LC mode in single LC cell, cannot be obtained. To develop the DFPG with the multi-directional LC alignment, we suggested two steps of the rubbing process supported by the photolithography process between each rubbing step. First, the LC alignment layer (polyimide, PI, SE-5811, Nissan Chemical Industries Co., Tokyo, Japan) was spin-coated on the patterned ITO electrode under the conditions of 1000 rpm for 5 s, 3000 rpm for 30 s, and 1000 rpm for 5 s. 

The coated PI layer was baked at 230 °C for 30 min and then it was uni-directionally rubbed with the roll-based rubbing machine at a roller speed of 300 rpm and a substrate speed of 10 mm/s in the horizontal direction, as shown in Figure 4b. Before the second rubbing process, the rubbed PI layer was protected by the photoresist (GXR-601) layer, and it was partially patterned by the photolithography process using the photo-mask, as shown in Figure 4c, where the LC alignment layer prepared for the ECB LC mode operation under the common slit was locally protected for the second rubbing process. With the partially passivated PI layer, the uncovered PI surface was rubbed orthogonal to the first rubbing direction, and then the residual GXR-601 layer was completely removed using acetone, as shown in Figure 4d, which schematically shows the final LC alignment directions of the top substrate of our DFPG, which has two orthogonal LC alignments for two LC domains of the ECB and TN LC modes. For our two domains of LC alignment, the photoresist process and its removing process should not physio-chemically degrade the LC alignment capability produced by the first rubbing process. Thus, the types of photoresist and their etchants should be carefully chosen, as mentioned in our experimental procedure. 

**Figure 5:**
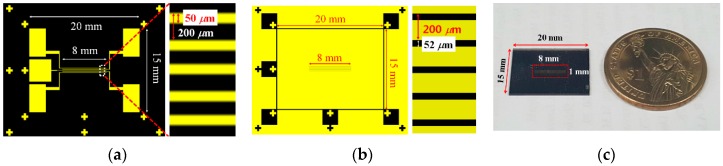
CAD images of photo-lithographic masks with the alignment marks for (**a**) ITO patterning and (**b**) Al slit patterning; (**c**) Image of the fabricated DFPG device.

Figure 5a,b show the photo-mask used for the ITO etching and the Al slit patterning process, respectively. In Figure 5a, five wide ITO patterns, directly connected to each ITO line pattern, can be seen, which were prepared for the wire-bonding process to control the electro-optic properties of the common slit and five switching slits individually with the driving signals. Figure 5c shows the finally implemented top substrate of the DFPG with the patterned ITO electrodes and the Al slit arrays. The substrate size was 15 × 20 mm^2^ in our sample implementation. The size of the actual area, which optically acts as the DFPG, was almost 8 × 1 mm^2^.

### 2.2. Theory of Depth Extraction

#### 2.2.1. Calculation of the Phase Modulation

**Figure 6:**
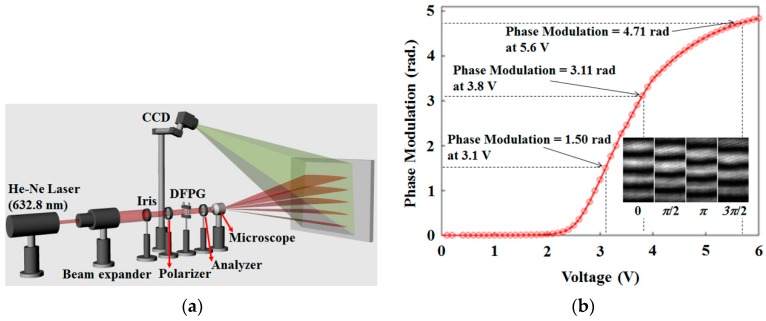
(**a**) Optical system for measuring phase modulation values of the common slit according to an applied voltage and their moving fringe patterns; (**b**) Measured phase modulation curve according to an applied voltage and the fringe patterns captured under Δφ = 0, π/2, π, and 3π/2 conditions.

When using our four-step phase-shifting scheme for the 3D depth extraction, the phase-shifting in our DFPG according to an applied voltage to the common slit needs to be precisely measured. The optical set-up of the phase modulation measurement is shown in Figure 6a. The coherent light source of a He-Ne laser (λ = 632.8 nm) was used to generate interference fringe patterns and to measure the field-dependent phase modulation of the LC layer operated by the ECB LC mode. The speckle noise and high order fringe visibility could be minimized to be a negligible level as shown in Figure 2 and Figure 6 because the coherence length of the He-Ne laser used in our experiment was 20 cm that was much smaller than those of coherent light sources used for conventional holographic interference experiments. The fringe visibility of the dynamic fringe patterns was about 0.9 in our system. This fringe visibility was enough to obtain the 3D depth extraction with our four-step phase-shifting and four steps of multi-spatial frequency scheme [21]. The beam from the He-Ne laser was expanded by a beam expander and was passed through an iris to reduce the optical noise so that a suitable spot size with a uniform beam intensity can cover all of the five Al slits. The light polarized with the *x*-axis polarizer was passed through the DFPG. The DFPG generated fringe patterns from two slits and the fringe patterns expanded by a projection lens were projected on a flat-panel screen. The projected fringe patterns on the flat-panel screen were captured by a CCD (FL2-14S3H, Pointgrey, Richmind, BC, Canada) with 4.4 µm pixel pitch and the field of view (FOV) of the CCD lens module was 12°.

We used the DFT method to measure the phase modulation. The fringe pattern by the DFPG was calculated using the equation *M_phase_* = *angle*[*X*(*pN*/Λ + 1)], as explained in our previous study, obtained using a mechanical moving slit system [21]. The values of the phase modulation were repeatedly calculated depending on the change of the applied voltage with an increase of 0.01 V per step. As shown in Figure 6b, the phase modulation over 3π/2 was achieved at 5.6 V. The voltage values for the four-step phase modulation were measured as 2.1, 3.1, 3.8, and 5.6 V, each for the phase shift of Δφ = 0, π/2, π, and 3π/2, respectively, as shown in Figure 6b. For each step of increasing the applied voltage to the common slit, the projected fringe patterns with the same spatial frequency were moved spatially by a quarter of the periodicity of the fringe patterns, as shown in the inset picture of Figure 6b.

**Figure 7:**
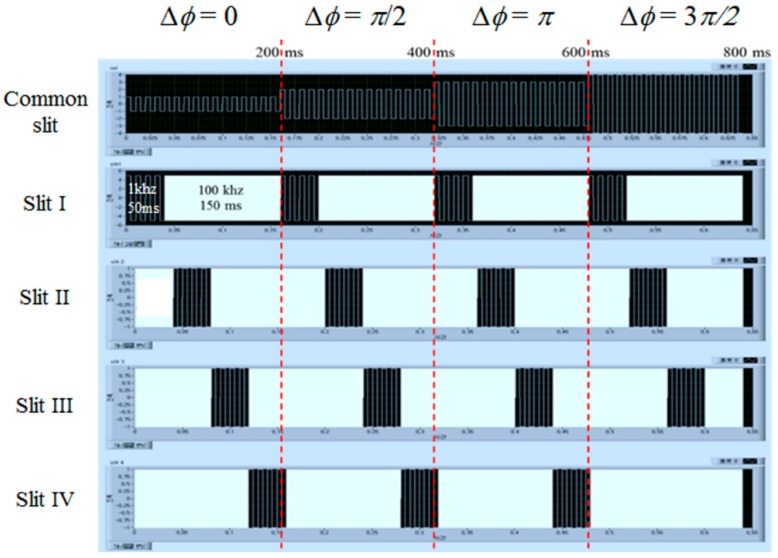
Driving waveforms of voltages applied to the LC layer on each slit of the DFPG utilizing the dual-frequency modulation for projecting 16 fringe patterns with the four different multi-spatial frequencies and the four-step phase shifting within the fast switching time.

Figure 7 shows the time sequence of the driving waveforms applied to the common slit part and four switching slit parts used for our DFPG. The driving waveforms for the common slit were changed every 200 ms and the amount of the applied voltage was increased to 2.1 V, 3.1 V, 3.8 V, and 5.6 V at 1 kHz to achieve phase shifting of Δφ = 0, π/2, π, and 3π/2, respectively, as shown in Figure 6. At a given phase-shifting amount condition, four-step spatial-frequency-varying fringe patterns were projected by applying dual-frequency-driving waveforms to the TN LC parts individually operated by the four patterned ITO electrodes. The turn-on waveform for each TN LC part of the switching slits was applied with 5 V of the applied voltage at 1 kHz for 50 ms, and then the turn-off waveform was applied with 5 V of the applied voltage at 100 kHz for 150 ms. These frequency-modulating AC waveforms were applied in sequence to the ITO electrode pattern of each switching slit every 50 ms to obtain four sets of fringe patterns with four different spatial frequencies. When applying the turn-off waveform with high frequency AC driving to the switching slit IV, the voltage required for the next step of the phase-shifting was applied to the ECB LC layer of the common slit and then, frequency-modulating waveforms were sequentially applied to the four TN LC layers to select four different spatial frequencies of the projected fringe patterns. In this manner, the whole scanning time required for the four-step multi-spatial frequencies and four-step phase shifting schemes could be completed in less than 800 ms. This means that the optical surface profilometry can capture 16 sets of fringe patterns distorted by an object in less than 800 ms. Our profilometry using the DFPG can exhibit quite fast scanning and capturing speeds suitable for hand-held dental applications, compared with our previous study of a 3D optical surface profilometry system constructed with a mechanically moving optical element [21].

#### 2.2.2. Theory of 3D Depth Extraction

**Figure 8:**
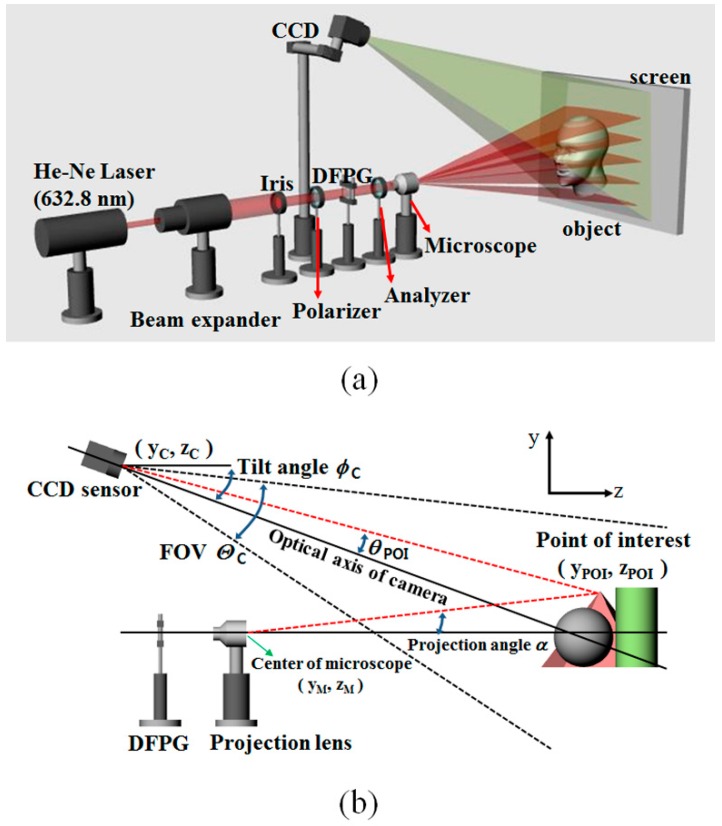
(**a**) Optical set-up to reconstruct the depth profile of a 3D object with the DFPG device; (**b**) Geometrical parameters of the 3D vision system used for 3D depth extraction.

Figure 8a shows the optical setup used to reconstruct a 3D depth profile with the DFPG device. The multiple sets of fringe patterns generated by the DFPG were projected though the projection lens onto the surface of an object. The fringe patterns distorted by the object surface were captured by a CCD. Figure 8b shows the geometrical parameters used to calculate the depth and position information from the CCD images. The optical system is composed of the DFPG, projection lens, CCD camera, and testing object. The DFPG and CCD are on the *y-z* plane. The center points of the projection lens, CCD, and object are defined by (*y_M_*, *z_M_*), (*y_C_*, *z_C_*) and (*y_POI_*, *z_POI_*), respectively. The fringe patterns are expanded through the projection lens, and the projected fringe patterns are distorted depending on the surface profile of an object. The optical axis of the DFPG is parallel to the *z*-axis, and the optical axis of the CCD is tilted at an angle of φ*_C_* to the *z*-axis. A point of interest (POI) on the object is expressed using geometrical parameters. As shown in Figure 8, a POI on the object is defined as (*y_POI_*, *z_POI_*). The line between the center of the projection lens and the POI is tilted at an angle of α with respect to the optical axis of the DFPG. The line between the center of the CCD and the POI is tilted at an angle of θ*_POI_* with respect to the optical axis of the CCD. In this case, the triangular method, defined by the geometrical parameters, leads to the following relationships:

(1) $$z_{POI}-z_{C}=-\left(y_{POI}-y_{C}\right)\cot\left(\phi_{C}-\theta_{POI}\right)\;\mathrm{and}$$

(2) $$z_{POI}-z_{M}=\left(y_{POI}-y_{M}\right)\cot\alpha$$

From Equations (1) and (2):

(3) $$z_{POI}=\frac{\left(y_{C}-y_{M}\right)-\left(z_{C}-z_{M}\right)\tan\alpha }{\tan\alpha -\tan\left(\phi_{C}-\theta_{POI}\right)}+z_{C}\;\mathrm{and}$$

(4) $$y_{POI}=\frac{\left(z_{C}-z_{M}\right)-\left(y_{C}-y_{M}\right)\cot\alpha }{\cot\alpha +\cot\left(\phi_{C}-\theta_{POI}\right)}+y_{C}$$

are obtained. Finally, Equations (3) and (4) show the depth and position information of the object calculated from the geometrical parameters [21].

## 3. Experimental Results and Discussion

### 3.1. Dynamic Phase Changing and Multiple Spatial Frequency Modulation Properties of the DFPG

**Figure 9:**
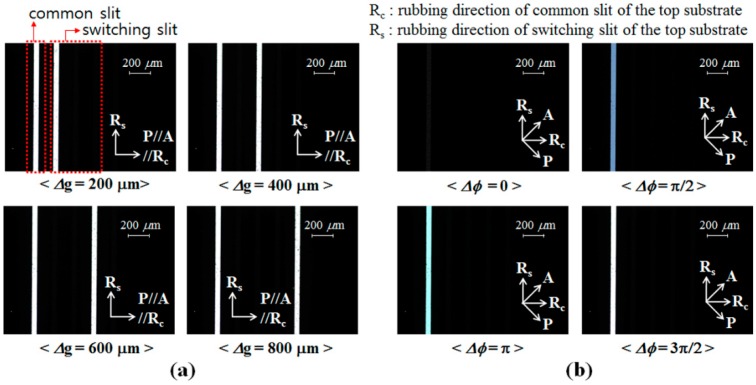
POM images depending on applied voltage conditions at each slit. (**a**) POM images measured between the parallel polarizers with varying selected switching slits for interference, which creates fringe patterns with four different spatial frequencies; (**b**) POM images showing the four-step phase shifting of the LC layer on the common slit captured between the crossed polarizers to show field-dependent birefringence variation.

In our DFPG for 3D optical surface profilometry, the transmission axes of the two polarizers and the rubbing direction of the LC alignment layer of the bottom substrate are aligned to be parallel to each other. In this device configuration, Figure 9a shows the polarization optical microscope (POM) images of the DFPG cell measured with applying the turn-on voltage to one of the TN LC layers used for switching the switching slits with varying the spacing for two beam interference, Δg = 200, 400, 600 and 800 µm. As shown in Figure 9a, the beam transmitted through the common slit, which is used as one beam spot for two-beam interference, is always under the turn-on state without intensity variation. However, the spacing between the two slits for interference can be controlled by the applied voltage conditions of the TN LC layers under the four switching slits. In all cases, three switching slit areas, which were not selected by the turn-on voltage, showed a dark texture without light transmittance.

To observe the field-dependent birefringence change in the ECB LC layer under the common slit, POM images of the DFPG cell were obtained between the crossed polarizers after detaching the parallel polarizers from the DFPG cell, as shown in Figure 9b. In this measurement, the POM images were captured by rotating the rubbing direction of the bottom substrate of the DFPG cell by 45° with respect to the crossed polarizers. On the common slit area, light transmittance or a color change could be observed owing to a variation of the phase modulation with increasing applied voltage to the ECB LC layer.

It is important to obtain the fast response property of the DFPG required in projecting 16 sets of multiple fringe patterns. The 3D depth profile is calculated from the 16 fringe pattern images distorted by surface morphology. The fast response time of each LC slit is positively necessary to improve the operating time of the 3D vision system. In this study, the dual-frequency LC was used to improve the response time of the LC layer switching. A dual-frequency LC exhibits the frequency-dependent dielectric anisotropy where Δε is positive under the low frequency operation and Δε is negative under the high frequency operation. That means that the LC molecules are reoriented along the applied field direction under the low frequency operation, whereas the LC molecules are reoriented perpendicular to the applied field direction under the high frequency operation.

**Figure 10:**
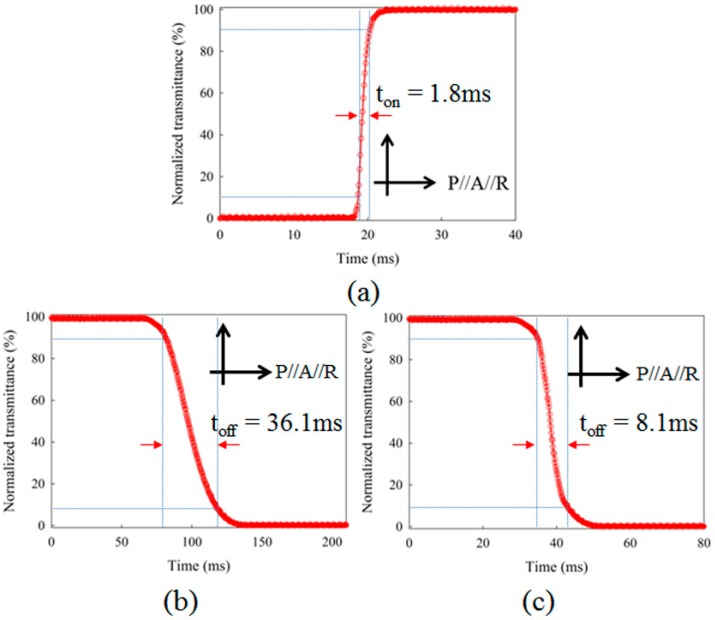
On/Off response times of the LC layer of the DFPG. (**a**) Field-on response time; (**b**) Field-off response time obtained by the natural relaxation of the LC layer due to the surface alignment effect without applying any electric field; (**c**) Off response time obtained by applying a high frequency field to the LC layer utilizing the frequency inversion effect of the LC dielectric anisotropy.

The response times of the DFPG were measured by electro-optic characterization equipment (LCMS200, Sesim Photonics Technology Co. Ltd., Uiwang, Korea). The common slit was blocked to measure the response times of the switching slits operated by the TN LC mode. Figure 10a shows that the turn-on response time switched by the low Hz operation of 5 V at 1 kHz was about 1.6 ms, sufficient for projecting multiple fringe patterns. However, the turn-off response time, measured by the field-off condition, showed a relatively slow response with a value of about 36.1 ms, as shown in Figure 10b. The natural field-off LC relaxation to the initial TN state is supported only by the LC elastic property and the LC surface anchoring, which was too slow to be used in our optical surface profilometry system. In our experiment, the turn-off response of the switching slit was improved by applying the high frequency operating field to obtain the field-driven turn-off property instead of the natural field-off LC relaxation. Figure 10c shows that the turn-off response was improved to about 8.1 ms by applying a high frequency AC field of 5 V at 100 kHz. Consequentially, the total response time of the TN slit was about 9.7 ms. As shown in Figure 7, the dual-frequency modulation scheme was used to switch four switching slits in sequence at a given phase-shifting amount of the common slit.

### 3.2. Depth Extraction from 3D Optical Profilometry

**Figure 11:**
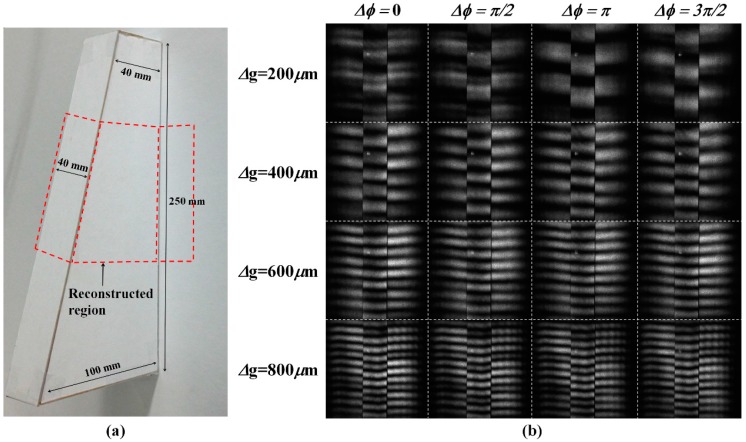
(**a**) Photographic image of the slanted object used for the 3D depth extraction; (**b**) CCD images of the distorted fringe patterns projected on the slanted object.

Figure 11a shows a the photographic image of the object used for the 3D depth extraction with the presented 3D optical surface profilometry system, which was deliberately chosen to have a continuously slanted surface together with an abrupt depth change discontinuity from the background reference surface. The smallest height at the abrupt side edges of the slanted object was over 40 mm, which was much higher than the optical wavelength used in our experiment. The regions marked with the dotted red box were reconstructed after projecting multiple sets of fringe patterns with our DFPG. Figure 11b shows the 16 sets of fringe patterns distorted on the slanted object. Four sets of laterally moved fringe patterns were obtained by changing Δφ via the common slit control. At a given Δφ condition, multiple fringe patterns having four different spatial frequencies were sequentially projected by selecting the Δg condition of the switching slits. Because there was a high depth discontinuity between the slanted surface and the background surface, some bright (or dark) fringe lines on the slanted surface were continuous with those on the background surface. This optical phase ambiguity could be solved using the four-step phase shifting scheme during the depth reconstruction [21,27].

**Figure 12:**
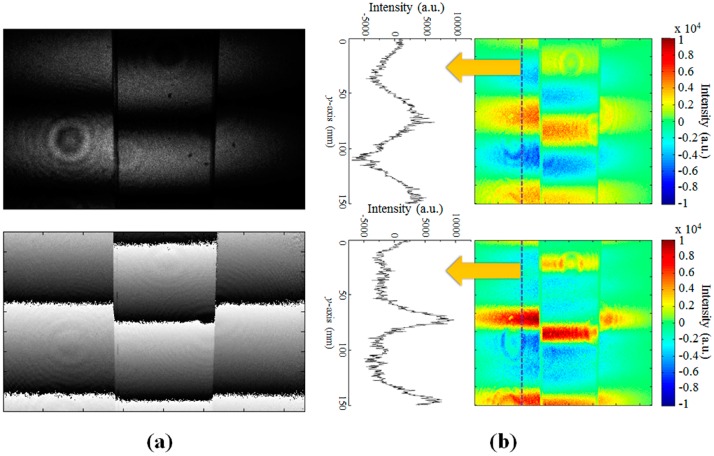
(**a**) Original fringe optical pattern image (upper image) projected on the slanted surface of Figure 11a using the interference slit of Δg = 200 µm and the synthesized phase map (lower image) obtained using the four-step phase shifting algorithm; (**b**) Fictitious scanning pattern images generated from two spatial frequencies (Δg = 200 and 800 µm) of the projected fringe patterns (the upper images) and four spatial frequencies (Δg = 200, 400, 600, and 800 µm) of the projected multiple fringe patterns (lower images).

The upper image of Figure 12a shows the enlarged CCD image of one of the projected fringe patterns, where we can observe some optical noises within the fringe patterns, which might be produced by some particles from our optical components. However, the synthesized phase map obtained after applying the four-step phase shifting algorithm, presented in the lower image of Figure 12a, shows that this type of the background optical noise within the fringe patterns can be successfully eliminated after the 3D depth reconstruction.

In our depth reconstruction, the fictitious scanning pattern is generated through multiple spatial frequencies of the interference fringes for the 3D depth reconstruction using the geometrical optical parameters [21,27]. In Figure 12b, the upper and the lower images show the fictitious scanning patterns generated from the two spatial frequencies of Δg = 200 and 800 µm and generated from full sets of four spatial frequencies (Δg = 200, 400, 600, and 800 µm), respectively. Compared with the upper image, the fictitious scanning pattern shown in the lower image can make much sharper peaks owing to more sinusoidal sets of the spatial frequencies used in the synthesis of the fictitious scanning pattern [21,27]. This could result in the enhanced depth resolution and precision after the 3D depth reconstruction. 

**Figure 13:**
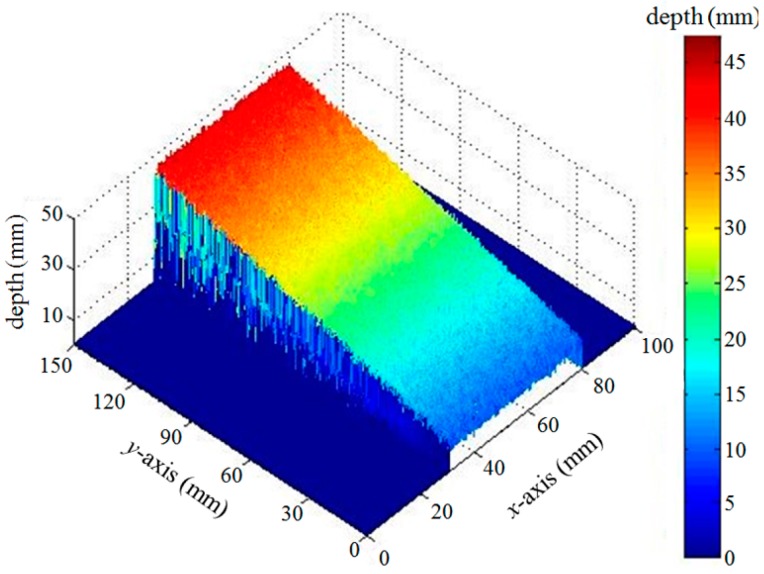
3D depth profile reconstructed from the slanted object.

Figure 13 shows the 3D depth profile reconstructed from the slanted object using the 16 sets of the dynamic fringe patterns. Compared with conventional phase unwrapping methods [28], one of the merits of the depth reconstruction based on the geometrical optical parameters used in our experiment is that the absolute depth values together with the 2D positional values, not just the relative phase values, can be obtained, as shown in Figure 13. 

When the size of an object to be measured with projecting the dynamic fringe patterns and to be reconstructed with the 3D depth extraction increases with approaching the values of the numerical aperture (NA) of the projection lens and the FOV of the CCD lens module, the calibrations of the fringe images captured by the CCD camera would be needed to reduce and to compensate the image distortions which might become more severe especially in the captured image boundaries owing to like the vignetting effect of the lens-based projection and imaging system. In our experiment, the NA of the projection lens was 0.4 and the FOV of the CCD lens module was 12°. Considering the projection distance of 1 m, the object size which can be measured in our experiment would be about 25 × 25 cm^2^. However, our object size was limited under 20 × 20 cm^2^ because we had a problem in enlarging the detectable object size because of the highly weakened fringe intensity with increasing the fringe pattern size as shown in Figure 2 and Figure 11. In our projection system, the much parts of the incident optical beams were blocked by the five slits and the intensity profiles projected by the projection lens decrease from the center to the image edges. We expect that this problems would be solved by improving the collimating projection lens part and by introducing the optical coupling module between the light source and the individual slits in the future work. However, this nonuniform intensity profile did not affect the depth extraction process in our four-step phase shifting and four steps of multi-spatial frequency scheme as shown in Figure 12. In our experiment set-up, we used the surface-distorted dynamic fringe patterns captured by the CCD lens module without the positional image calibration for the 3D depth extraction process. We checked the effect of the image distortion by our lens system by placing a grid pattern at the object plane, the image distortion was negligible under our experimental conditions of the FOV and the measurement distance. In Figure 13, the measurement error in the absolute value was less than 2 mm. This might originate from additional image distortions produced during the depth reconstruction process based on the geometric parameters, which are dependent on the perspective imaging condition expressed by the angle values of α and θ*_POI_* of Equations (3) and (4). We expect that the measurement accuracy can be further improved after calibration of these geometric parameters by using the periodic test patterns and the size of the measurable object can be enlarged after improving the projection parts and improving the CCD camera module.

**Figure 14:**
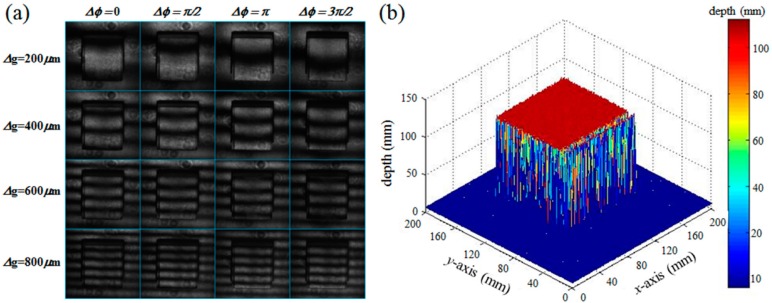
(**a**) CCD images of the distorted fringe patterns projected on the square box object; (**b**) 3D depth profile reconstructed from the square box object.

Figure 14a shows the CCD images of the distorted fringe patterns projected on a square box object (80 × 80 × 80 mm^3^). The perspective view of the 3D depth profile obtained after the 3D depth reconstruction is presented in Figure 14b with the absolute coordinate axis values. For each *x*, *y*, and *z* coordinate axis, the measurement errors of the extracted 3D depth profile were 1.6, 0.9, 1.2 mm, respectively, which were slightly different depending on the coordinate axis. This coordinate-dependent measurement error might originated in the different perspective viewing conditions between the projection lens part and the image-capturing CCD camera part in our optical system as shown in Figure 8. In the previous approaches, most of the optical surface profilometry systems are based on the phase unwrapping algorithm for the depth reconstruction [28,29,30] and need more complex optical elements like the time-sequentially switching multi-wavelength light sources [31,32], additional optical components [33,34], and a mechanical translation state [21,35] for the projection to generate multi-spatial-frequency or/and phase-shifting fringe patterns. Our optical surface profilometry system based on the electrically switching single DFPG cell can generate dynamic fringe patterns in a fast switching time and can extract the depth and 2D positional information of a 3D object even for cases having large surface depth discontinuities.

## 4. Conclusions

3D optical surface profilometry systems have been applied to measure the depth profile of a 3D object and their application fields are much growing recently. To enhance the accuracy of the 3D depth extraction and/or to avoid the phase ambiguity problem, multiple sets of fringe pattern projections are essential in the optical surface profilometry system. We suggested a single LC cell DFPG operated by a simple passive driving scheme, which can be fabricated into a compact optical module. To project 16 sets of dynamic fringe patterns having the optical features of four-step phase shifting and four different spatial frequencies without any mechanical parts and any complex and expensive SLM parts, two types of LC modes—the ECB LC mode and the TN LC mode—were included in the single LC cell by preparing two orthogonal LC alignments on one of the LC alignment layers. Five optical slits were prepared using photolithography and aligned with the patterned LC alignment layer, where one common slit with the ECB LC layer was used for the four-step phase-shifting control and four switching slits with the TN LC layers were used for the generation of four different spatial frequencies. Moreover, to improve the scanning time required for generating the 16 sets of dynamic fringe patterns, dual-frequency switching LC was employed, where the switching time—including the turn-on and the turn-off times—controlled by the frequency modulation increased four-fold about four times compared with the switching time controlled by the conventional single frequency. As a result, the 16 sets of fringe patterns could be projected and captured within 800 ms. For the depth reconstruction procedures, the phase shifting in the common slit of the DFPG according to the applied voltages was accurately measured. The resolution of the depth reconstruction obtained by applying the DFT method and the optical geometric parameters was improved by using the four different sets of spatial frequencies of the dynamic fringe patterns. Our 3D optical surface profilometry system yields 3D depth profiles with respect to the absolute value 3D coordinates, not just the relative phase depth maps, even for objects with abruptly changing surface profiles. Owing to the compactness and fast switching properties of the DFPG, we expect that the presented 3D optical surface profilometry system can be applied to a hand-held 3D vision module, which can be widely used for several mobile applications requiring 3D depth information.

## Abbreviations

The following abbreviations are used in this manuscript:

DFPG	Dynamic Fringe Pattern Generator
LC	Liquid Crystal
TN	Twisted Nematic
ECB	electrically controlled birefringence
ITO	indium thin oxide
He-Ne Laser	Helium-Neon Laser
DFT	Discrete Fourier Transform
POM	Polarizing Optical Microscope
FOV	Field of View
